# Construction of Improved Tools for Protein Localization Studies in *Streptococcus pneumoniae*

**DOI:** 10.1371/journal.pone.0055049

**Published:** 2013-01-22

**Authors:** Mafalda X. Henriques, Maria João Catalão, Joana Figueiredo, João Paulo Gomes, Sergio R. Filipe

**Affiliations:** 1 Laboratory of Bacterial Cell Surfaces and Pathogenesis, Instituto de Tecnologia Química e Biológica, Universidade Nova de Lisboa, Oeiras, Portugal; 2 Department of Infections Diseases, National Institute of Health, Lisbon, Portugal; Julius-Maximilians-University Würzburg, Germany

## Abstract

We have constructed a set of plasmids that allow efficient expression of both N- and C-terminal fusions of proteins of interest to fluorescent proteins mCherry, Citrine, CFP and GFP in the Gram-positive pathogen *Streptococcus pneumoniae*. In order to improve expression of the fluorescent fusions to levels that allow their detection by fluorescence microscopy, we have introduced a 10 amino acid tag, named i-tag, at the N-terminal end of the fluorescent proteins. This caused increased expression due to improved translation efficiency and did not interfere with the protein localization in pneumococcal bacteria. Localizing fluorescent derivatives of FtsZ, Wzd and Wze in dividing bacteria validated the developed tools. The availability of the new plasmids described in this work should greatly facilitate studies of protein localization in an important clinical pathogen.

## Introduction

Studies on the sub-cellular localization of bacterial proteins have changed our view on the organization of bacterial cells. Initially, these studies were essentially restricted to the model organisms *Escherichia coli* and *Bacillus subtilis*. Despite their importance, these organisms do not eliminate the need for specific studies using different clinically important bacterial species, given the diversity in terms of morphology, physiology and metabolism among bacteria. Therefore, we have recently witnessed the development of new tools to allow cell biology studies in different pathogenic bacteria [1], [2].

*Streptococcus pneumoniae* is a major cause of morbidity and mortality worldwide. This Gram-positive bacterium is associated with a range of infections, which can vary from simple otitis media to more complicated ones, such as pneumonia or meningitis. Infection by this important human pathogen is of particular concern in developing countries, in which pneumococcal septicemia causes 25% of all preventable deaths in children under the age of five [3].

In order to design new and more efficient strategies to fight pneumococcal infections it is essential to understand how these bacteria divide or perform specific tasks important for their survival inside the host, such as the synthesis of peptidoglycan, the target of beta-lactam antibiotics which are widely used against *S. pneumoniae*, or the synthesis of the capsular polysaccharide, the target of several successful anti-pneumococcal vaccines. An important step to accomplish this goal is the study of the localization of proteins involved in these processes. However, for a long time, cell biology studies in *S. pneumoniae* were limited by the lack of appropriate tools. Localization of pneumococcal proteins involved in cell wall synthesis [4],[5] and cell division [6], [7], [8] was initially accomplished using immunofluorescence techniques, which require cell fixation and lysis to allow access of the antibodies to the target proteins. Therefore, immunofluorescence can not be used with live cells and is prone to generate artifacts [9]. It was only recently that the first studies on the localization of proteins in live pneumococcal cells, using fluorescent protein fusions, tagged to Green Fluorescent Protein (GFP), was reported [1]. Since then, other proteins involved in processes such as cell division [10], [11], cell wall synthesis [12] and capsular polysaccharide synthesis [13], [14] have been localized in live pneumococcal cells. However, the variety of tools available for these studies is still limited.

In this paper, we report the construction of new plasmids that expand the tools available for *S. pneumoniae* cell biology studies by allowing the expression of N- or C- terminal protein fusions to different fluorescent reporters, namely mCherry, Citrine, CFP and GFP. For this purpose we have improved the expression of the various fluorescent proteins in *S. pneumoniae*, by introducing an upstream tag, named “i-tag”, which increases protein translation. The availability of these plasmids should greatly facilitate studies of protein localization in this important clinical pathogen.

## Results and Discussion

### Expression of mCherry, Citrine, CFP and GFP in *S. pneumoniae*

**Figure 1:**
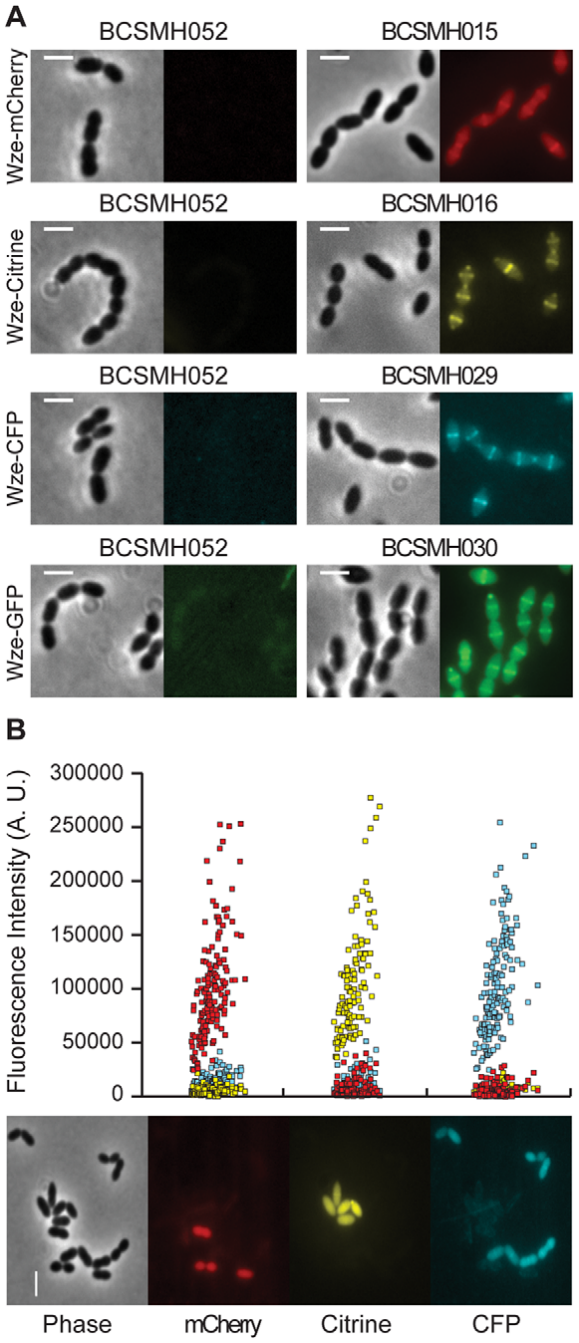
Fluorescent signals emitted by mCherry, Citrine, CFP and GFP protein fusions are detectable in live *S. pneumoniae* bacteria. (A) The septal localization of Wze protein fusions expressed in the encapsulated ATCC6314 strain is shown for Wze-mCherry (strain BCSMH015), Wze-Citrine (strain BCSMH016), Wze-CFP (strain BCSMH029), Wze-GFP (strain BCSMH030). No comparable signal was detected in cells containing an empty vector (strain BCSMH052) visualized using appropriate filters for each fluorescent protein. (B) Three cultures of unencapsulated R36A strain expressing Wze-mCherry (strain BCSMH006, red dots), Wze-Citrine (strain BCSMH007, yellow dots) and Wze-CFP (strain BCSMH035, blue dots) were prepared for fluorescence microscopy observation as described in the Materials and Methods section, mixed on the same slide, and visualized using appropriate filters for each fluorescent protein. Fluorescence intensity was plotted, showing that the signals from each protein do not overlap. A representative image is shown at the bottom of the figure. Exposure times were 5 sec. Scale bar: 2 µm.

*S. pneumoniae* is a microaerophile organism and therefore can only grow in the presence of low levels of oxygen, which may impair the correct folding of GFP-like proteins that are known to require the presence of oxygen [15]. We have expressed fusions of Wze, a protein required for the regulation of the synthesis of the capsule polysaccharide [3], to four different fluorescent proteins, mCherry [16], Citrine [17], CFP [18] and GFP [19], two of which (CFP and GFP) had not been previously used in *S. pneumoniae*. The protein fusions Wze-CFP (BCSMH029) and Wze–GFP (BCSMH030), expressed in the encapsulated strain ATCC6314, allowed the visualization of Wze protein at the septum (Fig. 1A), in accordance to what we have previously described for Wze-mCherry (BCSMH015) and Wze-Citrine (BCSMH016) [14]. This indicates that sufficient amounts of fluorescent proteins tested were able to fold correctly, allowing detection, in the growth conditions used.

In order to test which of the four fluorescent proteins could potentially be used together for applications that require co-localization studies in *S. pneumoniae*, we mixed and analyzed various combinations of strains expressing the fluorescent proteins (data not shown). Fluorescence microscopy analysis of a slide containing a mixture of three different cultures of the unencapsulated laboratory R36A strain expressing Wze-mCherry (BCSMH006), Wze-Citrine (BCSMH007) and Wze-CFP (BCSMH035), in the cytoplasm (Fig. 1B), showed that the fluorescent signals of these three proteins did not overlap. We further confirmed that the spectral overlap of CFP, Citrine and mCherry could be limited with the filter sets used (Fig. S1).

**Figure 2:**
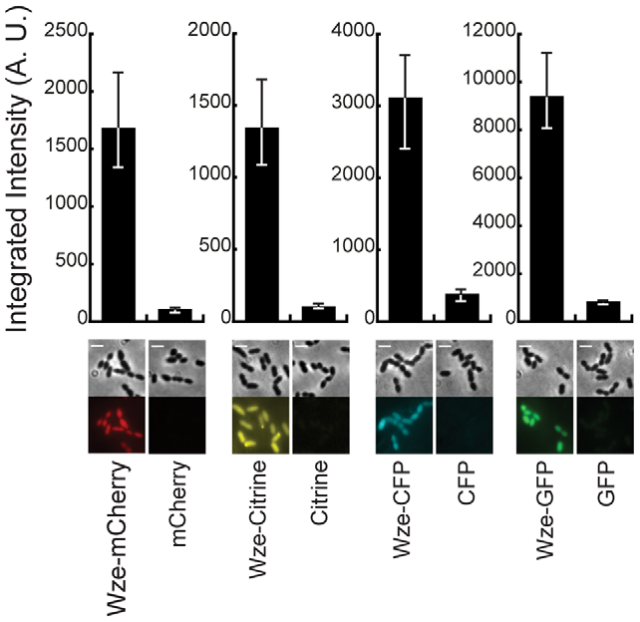
Fluorescent signals emitted by untagged mCherry, Citrine, CFP and GFP proteins are not detectable. The median intensity, with 25% (white error bars) and 75% (black error bars) inter-quartile range (in arbitrary units) of the fluorescence signal in unencapsulated bacteria expressing Wze-mCherry (strain BCSMH006), mCherry (strain BCSMH032), Wze-Citrine (strain BCSMH007), Citrine (strain BCSMH033), Wze-CFP (strain BCSMH033), CFP (strain BCSMH034), Wze-GFP (strain BCSMH035) and GFP (strain BCSMH036), is plotted. At least 100 cells of each strain were quantified. Representative images of each strain are shown at the bottom. Scale bar: 2 µm.

We then constructed new vectors for cell biology studies in *S. pneumoniae* (plasmids pBCSMH001, pBCSMH002, pBCSMH018 and pBCSMH020) expressing the four fluorescent proteins, not fused to any protein. These replicative vectors are derivatives of the plasmid pLS1, which is reported to be at a copy number of 24 per pneumococcal bacteria [20]. Surprisingly, all strains expressing solely the untagged fluorescent proteins mCherry (BCSMH032), Citrine (BCSMH033), CFP (BCSMH034) and GFP (BCSMH036) showed extremely low levels of fluorescence (Fig. 2).

### Optimization of expression of fluorescent proteins in *S. pneumoniae*

**Figure 3:**
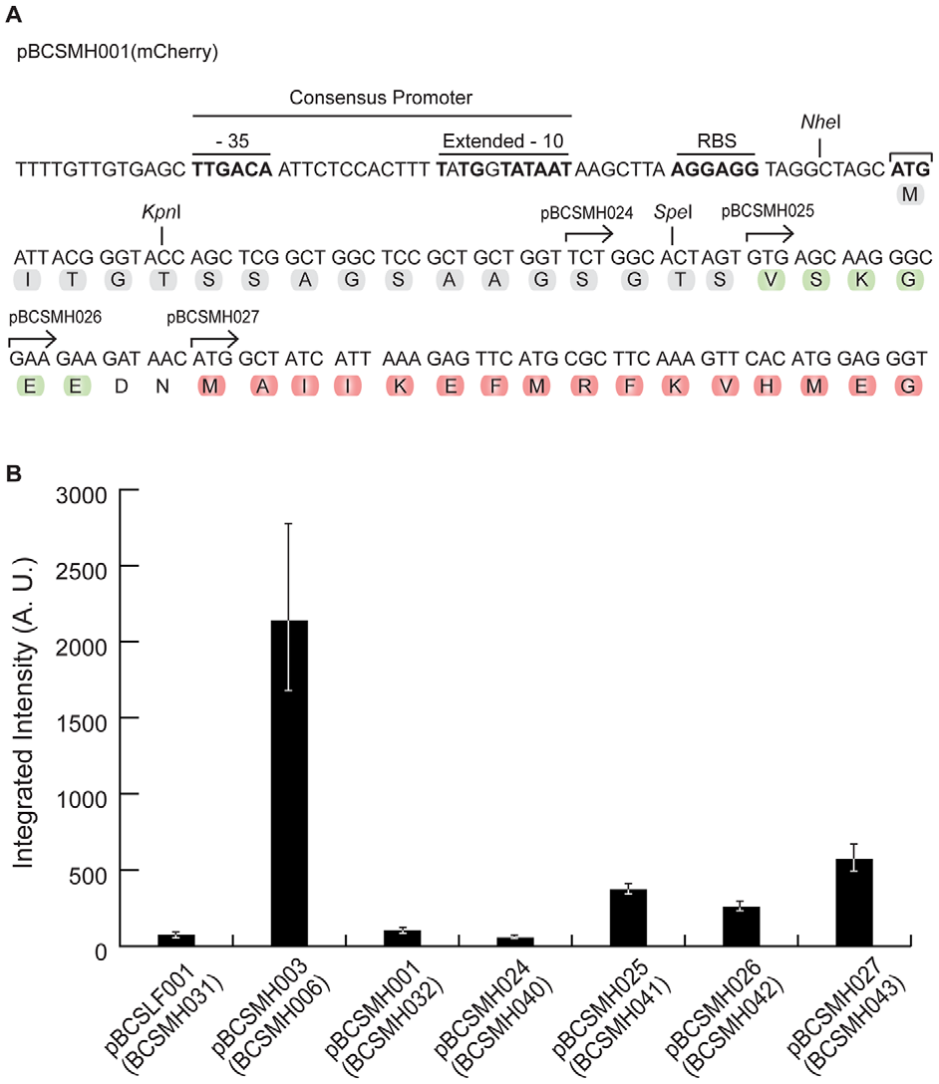
Linker region present at the N-terminal of fluorescent proteins does not impair expression of fluorescence. (A) Partial sequence of plasmid pBCSMH001 highlights the linker region (grey), the GFP-like termini region (green) and the encoded mCherry (red). Amino acids between the first methionine and the aminoacids indicated by the arrows were removed to generate plasmids pBCSMH024-027 encoding truncated forms of mCherry. (B) The median intensity, with 25% (white error bars) and 75% (black error bars) inter-quartile range (in arbitrary units) of the fluorescence signal obtained from strains expressing Wze-mCherry (strain BCSMH006), truncated mCherry forms (strains BCSMH040–043) and mCherry alone (strain BCSMH032) is plotted. At least 100 cells of each strain were quantified.

The four fluorescent proteins used in our studies all included, at their N-terminal, a linker region, which introduced a spacer between the fluorescent protein and the protein of interest, and a GFP-like terminus region that had been proposed to stabilize the fluorescent signal [16] (Fig. 3A). We therefore asked whether this N-terminal linker was responsible for the lack of fluorescence in *S. pneumoniae*. To answer this question we constructed a series of plasmids, encoding mCherry or Citrine, in which successive fragments of the linker were removed, leaving intact the original promoter region and starting codon (Fig. 3A). Quantification of the fluorescent signal expressed by pneumococcal strains encoding truncated mCherry proteins (strains BCSMH040 to BCSMH043) showed that removal of the linker caused only a small increase in the observed fluorescence (Fig. 3B), which was still 80% lower than the signal obtained for strain BCSMH006, expressing Wze-mCherry [14]. No increase in the fluorescence signal was observed when the linker region was removed from the Citrine protein (data not shown) indicating that this region was not impairing fluorescence in pneumococcal bacteria.

**Figure 4:**
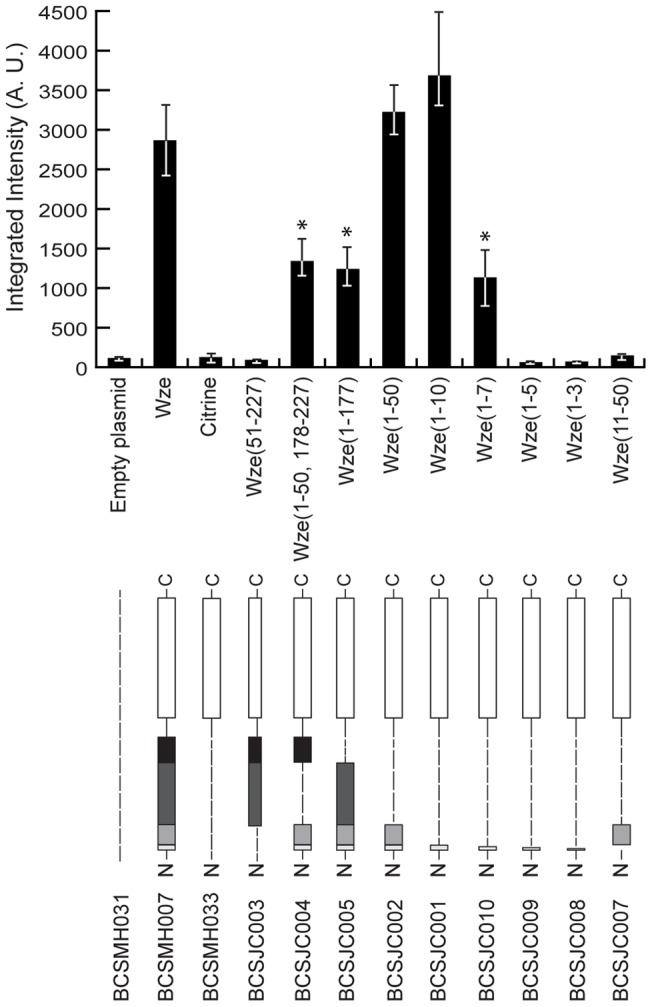
The first ten amino acids of Wze are required to obtain high levels of fluorescence. (Upper panel) Median fluorescence, with 25% (white error bars) and 75% (black error bars) inter-quartile range (in arbitrary units) emitted by Citrine fused to specific aminoacid sequences for Wze, as indicated below the graphic, in *S. pneumoniae* R36A strain. Strain BCSMH031 was used as a negative control. At least 100 cells of each strain were quantified. Exposure time was 5 sec. Kruskal-Wallis analysis with Dunn's multiple post-test comparison did not reveal significant differences between the BCSMH007 strain, expressing Wze-Citrine, and strains BCSJC001 and BCSJC002, expressing Citrine constructs that still carry the first 10 aminoacids of Wze (P>0.05). A significant reduction of the fluorescent signal was observed in strains BCSJC004, BCSJC005 and BCSJC010 (*, P<0.01). (Bottom panel) A schematic representation of each Wze constructs is shown. White box represent the Citrine protein while gray boxes represent different regions of Wze protein (lighter grey – aminoacids 1 to 10, light grey – remaining N-terminus region between aminoacids 11 to 50, dark grew – central region between aminoacids 51–176, black box – C-terminus region between aminoacids 178–227. Strain name are indicated below.

As a second hypothesis, we asked whether there was a specific nucleotide sequence present in the coding sequence of Wze that increased protein expression in the Wze-Citrine fusion. This was based on the fact that the 5′ coding region of the mRNA, immediately after the start codon, has been proposed to have an important role in ensuring protein translation in bacteria. This effect seems to be more dependent on the predicted mRNA secondary structure of this region than on codon usage bias or GC contents [21]. In order to identify that putative nucleotide sequence, pneumococcal bacteria were transformed with plasmids encoding different truncated forms of Wze-Citrine fusion (Fig. 4). Deletion of the central region (amino acids 51–177, strain BCSJC004) or the C-terminal region (amino acids 178–227, strain BCSJC005) of Wze resulted in proteins that were still fluorescent, although a significant decrease of about 40% in the intensity of the fluorescence signal was observed in both cases (Fig. 4). However, when the first 50 amino acids of the N-terminal region of Wze were deleted, in strain BCSJC003, the expression of fluorescence was completely lost (Fig. 4).

In order to determine the minimum size of the N-terminal region of Wze required for expression of fluorescence, *S. pneumoniae* R36A strain was transformed with plasmids encoding the first 3, 5, 7, 10 or 50 aminoacids of Wze, linked to the N-terminal of Citrine, and analyzed by fluorescence microscopy. Strains BCSJC008 and BCSJC009, expressing the Citrine protein fused to the first 3 or 5 amino acids of Wze, respectively, showed no fluorescence (Fig. 4). Strain BCSJC010, expressing the Citrine protein fused to the first 7 amino acids of Wze, showed a significant reduction in the fluorescent signal relative to that observed with the expression of the entire Wze-Citrine protein in strain BCSMH007 (Fig. 4). However, strains BCSJC001 and BCSJC002, expressing the Citrine protein fused to the first 10 or 50 amino acids of Wze, respectively, showed fluorescence levels similar to that obtained for strain BCSMH007, expressing the original Wze-Citrine fusion (Fig. 4). Expression of Wze aminoacids 11 to 50 fused to Citrine did not result in a fluorescent protein (Fig. 4), confirming that the first 10 amino acids of Wze were necessary and sufficient for expression of Citrine in *S. pneumoniae*. We have named this 10 amino acid tag, which improved protein expression in pneumococcal bacteria, “i-tag”.

**Figure 5:**
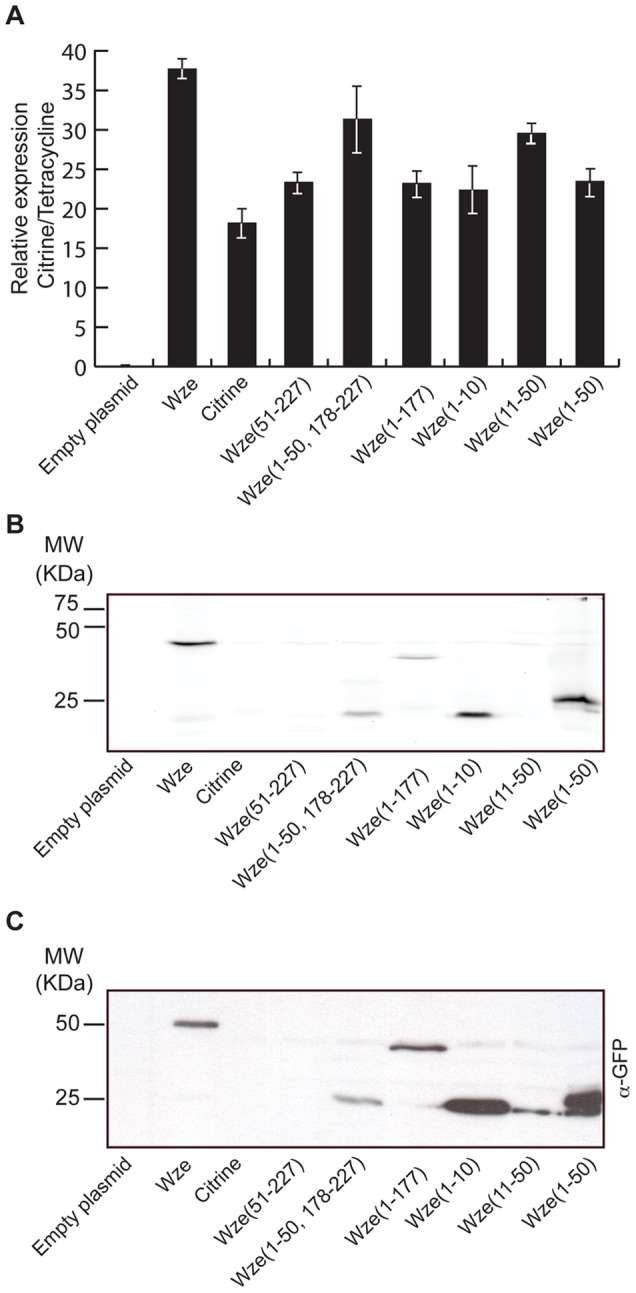
Increased fluorescence resulting from the presence of the “i-tag” is due to increased protein levels. (A) mRNA encoding Citrine was quantified by Real-time PCR in strains expressing specific aminoacid sequences from Wze fused to Citrine, relatively to the mRNA for the tetracycline resistance protein which is encoded in the plasmid backbone. Strains analyzed were BCSMH031 (transformed with an empty vector), BCSMH007 (expressing full Wze-Citrine), BCSMH033 (expressing Citrine), BCSJC003 (expressing Wze_(51–227)_-Citrine), BCSJC004 (expressing Wze_(1–50, 178–227)_-Citrine), BCSJC005 (expressing Wze_(1–177)_-Citrine), BCSJC001 (expressing Wze_(1–10)_-Citrine), BCSJC007 (expressing Wze_(11–50)_-Citrine) and BCSJC002 (expressing Wze_(1–50)_-Citrine). Cell extracts from these strains were separated by SDS-Page and analyzed using a Fluorescent Image Analyzer (B) and by Western-blot analysis using an antibody that recognizes Citrine protein (C), showing that fluorescence in strains containing the i-tag is due to increased protein levels and not to increased mRNA levels.

The increased fluorescence due to the presence of the i-tag fused to Citrine could be the result of higher mRNA or higher protein levels. We therefore quantified, by real-time PCR, the levels of mRNA encoding untagged Citrine, Wze-Citrine fusion and the various truncated forms of this fusion, in exponentially growing bacteria, relatively to the mRNA for the tetracycline resistance marker, encoded in the plasmid backbone. Fig. 5A shows that levels of the different mRNAs were not sufficiently different to explain the variability in fluorescence expression. However, analysis of Citrine protein levels in the same strains showed a correlation between strains in which Citrine protein could be detected and strains which were fluorescent, namely those encoding for Wze-Citrine (strain BCSMH007) and all that included the first 10 amino acids of the Wze fused to Citrine (strains BCSJC001, BCSJC002, BCSJC004 and BCSJC005). Taken together these results show that fusion of the i-tag to Citrine increased fluorescence levels due to increased protein levels and not increased mRNA levels.

**Figure 6:**
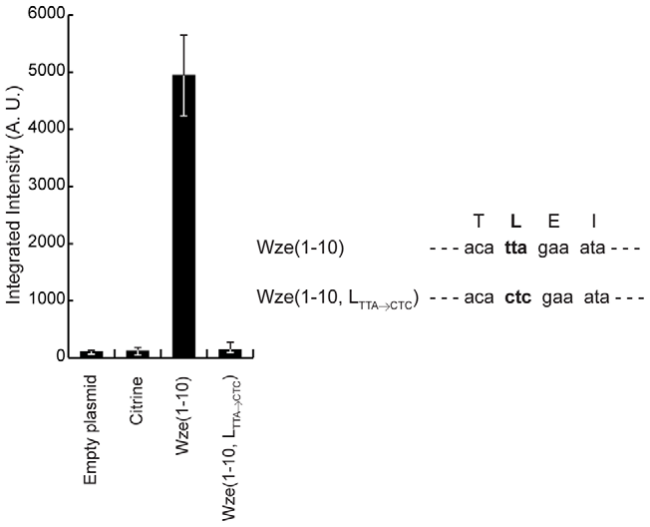
The nucleotide sequence of the i-tag determines the expression of fluorescence. (Left panel) Median fluorescence, with 25% (white error bars) and 75% (black error bars) inter-quartile range (in arbitrary units) of the fluorescence emitted by Citrine from the following strains: BCSMH031 (empty plasmid), BCSMH033 (expressing Citrine), BCSJC001 (expressing Wze_(1–10)_-Citrine), BCSJC006 (expressing Wze_(1–10)*_-Citrine in which the sequence encoding for Leucine was changed from TTA to CTC). At least 100 cells of each strain were quantified. (Right panel) Sequence encoding aminoacids 3–6 of Wze present in Wze_(1–10)_-Citrine and Wze_(1–10)*_-Citrine.

To determine if increased protein levels resulted from higher translation rates or increased protein stability, we generated a silent mutation in the sequence encoding the i-tag, fused to Citrine, and analyzed the fluorescence of the resulting constructs. We were able to identify mutations that did not alter the amino acid sequence of the tag, but resulted in loss of fluorescence, namely the substitution of UUA leucine codon by CUC (Fig. 6). Given that protein sequence was not altered, we can rule out the hypothesis that the i-tag acted by increasing stability of the fusion proteins. We also do not think that increased expression is due to the introduction of an additional ribosome-binding site, as previously reported by Halfmann and colleagues [22], as we did not introduce any additional sequence upstream of the starting codon. Therefore the presence of the nucleotide sequence encoding the i-tag results in increased translation rates, possibly by destabilizing the mRNA structure of this region and thus facilitating ribosome binding to the mRNA molecule.

### Construction of plasmids for the expression of fluorescent protein fusions in *S. pneumoniae*

**Figure 7:**
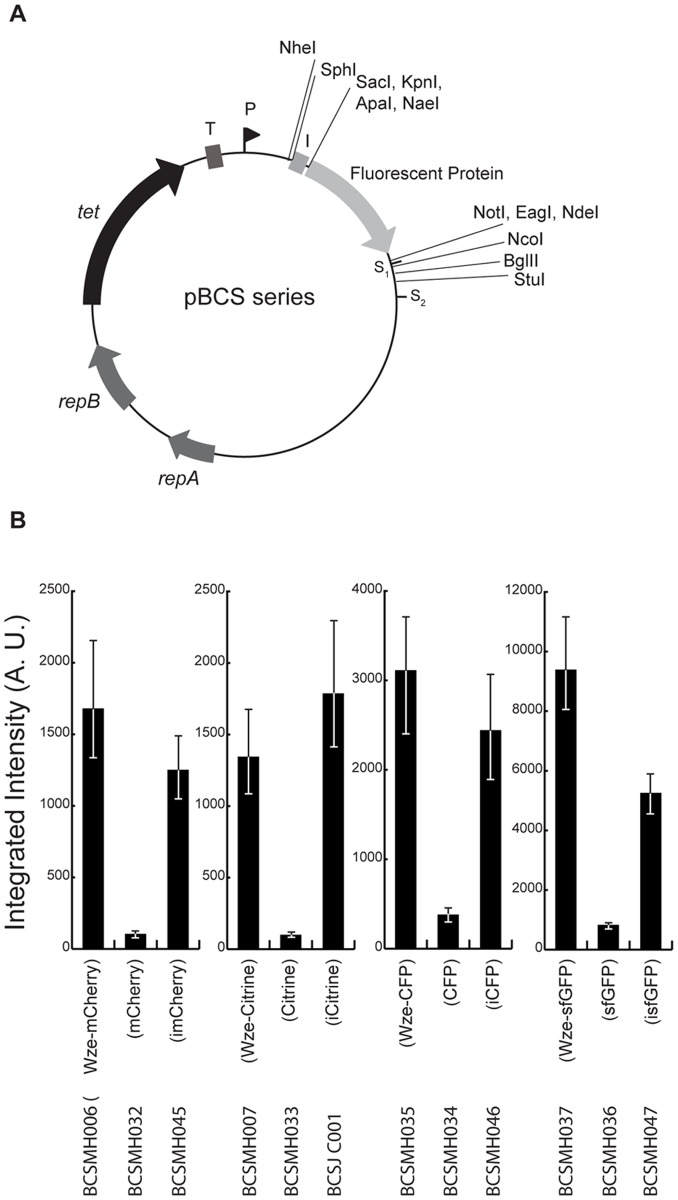
New plasmids for *S. pneumoniae* cell biology studies. (A) Map of the pBCS plasmids. Fluorescent protein refers to mCherry, Citrine, CFP or GFP, encoded by plasmids pBCSMH030, pBCSJC001, pBCSMH031 and pBCSMH032, respectively. *Apa*I and *Nae*I restriction sites, highlighted with an asterisk, are not available in plasmid pBCSMH030. *repA, repB*, plasmid replication genes. *tet*, tetracycline resistance marker. T, transcription terminator. P, promoter. S_1_, stop codon in plasmid pBCSMH030. S_2_, stop codon in plasmids pBCSJC001, pBCSMH031 and pBCSMH032. (B) Comparison of fluorescence emitted by strains expressing mCherry, Citrine, CFP and GFP alone, their improved i-tag versions and their Wze fusions. The median fluorescence, with 25% (white error bars) and 75% (black error bars) inter-quartile range (in arbitrary units) is plotted. At least 100 cells of each strain were quantified. Strain names are indicated below.

We have redesigned plasmids pBCSMH001, pBCSMH002, pBCSMH018 and pBCSMH020, expressing untagged fluorescent proteins (Fig. 2) to improve expression of mCherry (pBCSMH030), Citrine (pBCSJC001), CFP (pBCSMH031) and GFP (pBCSMH032) by including the “i-tag” upstream of the fluorescent proteins (Fig. 7A). Additionally, we have introduced unique restriction sites flanking the genes encoding for the fluorescent proteins, so that the resulting plasmids can be used to express both N- and C-terminal fluorescent fusions of *S. pneumoniae* proteins, under the control of a constitutive consensus *S. pneumoniae SigA* promoter [23]. Analysis of strains containing these new plasmids showed that expression of i-tagged fluorescent proteins generates cells with fluorescence levels comparable to the ones expressing the Wze-fusions (Fig. 7B). Importantly, the i-tagged fluorescent proteins were dispersed throughout the cytoplasm of the encapsulated pneumococcal cells (Fig. S2)) indicating that the i-tag did not interact with Wzd or Wze proteins, normally localized at the dividing septum of encapsulated strains [14].

**Figure 8:**
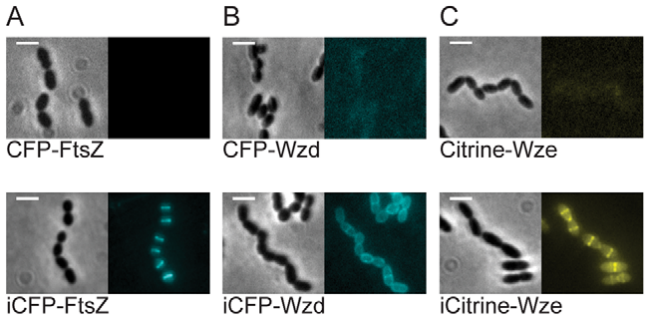
Applications of the developed tools for localization of *S. pneumoniae* proteins. (A) Localization of the cell division protein FtsZ as a N-terminal fusion to CFP (CFP-FtsZ, strain BCSMH050) and to i-tagged CFP, (iCFP-FtsZ, strain BCSMH051). (B) Localization of the membrane Wzd protein as a N-terminal fusion to CFP (CFP-Wzd, strain BCSJF004) and to improved i-tagged CFP, (iCFP-Wzd, strain BCSJF003). (C) Localization of the Wze tyrosine kinase as a N-terminal fusion to Citrine (Citrine-Wze, strain BCSJF002) and to improved i-tag Citrine (iCitrine-Wze, BCSJF001). The i-tagged versions of the fluorescent reporters allowed the visualization of each protein at the expected sub-cellular region of bacteria, the division septum. Exposure times: 5 sec. Scale bar: 2 μm.

The developed tools were used to localize different proteins in *S. pneumoniae*, namely FtsZ, a central protein for bacterial division; Wzd, a membrane protein, and Wze, a cytoplasmic tyrosine kinase, both of which localize at the bacterial division septum when expressed together in pneumococcal cells. Figure 8 shows that fusions of these proteins to the C-terminal of CFP or Citrine only produced a fluorescent signal when the improved versions, containing the “i-tag”, of the fluorescent proteins were used, and not with the normal untagged versions. It should be highlighted that these transformants also express from the native chromosomal locus, the non-fluorescent versions of the FtsZ, Wzd or Wze proteins, presumably increasing the cellular concentration of these proteins. Although we have not seen any difference in the localization pattern of fluorescent derivatives of Wzd-Citrine or Wze-Citrine expressed in the absence or in the presence of the non-fluorescent original proteins [14], this may not be the case for fluorescent derivatives of other pneumococcal proteins.

### Final remarks

We were able to significantly improve the expression of four fluorescent proteins, mCherry, Citrine, CFP and GFP in the Gram-positive bacteria *S. pneumoniae*, by designing a tag that increases translation efficiency of heterologous proteins. The set of plasmids encoding improved versions of these fluorescent proteins allows the expression of both N- and C-terminal fluorescent fusions of pneumococcal proteins and should greatly facilitate cell biology studies in this important pathogen.

## Materials and Methods

### Bacterial strains and growth conditions

Bacterial strains and plasmids used in this study are listed in Table S1). *S. pneumoniae* was grown in C + Y liquid medium [24] at 37°C, without aeration, or in tryptic soy agar (TSA, Difco) plates supplemented with 5% sheep blood (Probiologica). Tetracycline was added to the media at 1 µg/ml final concentration.

### DNA manipulation procedures

*S. pneumoniae* competent cells preparation and transformation was executed as previously described [25]. PCR products and plasmid DNA were purified with kits Wizard® SV Gel and PCR Clean-up System and Wizard® Plus SV Minipreps, respectively (Promega). PCR fragments were amplified with Phusion High-fidelity DNA polymerase (Finnzymes). Restriction enzymes were from New England Biolabs. Primers used in this study are listed in Table S2).

### Construction of plasmids for protein expression in *S. pneumoniae*

For expression of CFP and GFP, not fused to any protein, we constructed plasmids pBCSMH018 and pBCSMH020 by amplification of the CFP and GFP(P5) coding sequences from plasmids pMUTIN-CFP and pTrc99A-GFP, respectively, with primers 1/2 and primers 7/8, followed by restriction and ligation to the vector backbone obtained by amplification of pBCSMH001 with primers 3 and 4. The GFP version used in this work is the GFP(P5), reported by Fischer and DeLisa [19], which carries several mutations compared to GFP+ (K26R, T65A, V68L, S72A, R80Q, S99F, T153K, A163V, A206V, V219I) and has an improved folding kinetic and slower unfolding.

For expression of Wze-CFP and Wze-GFP, the *wze* gene was amplified with primers 5 and 6 and cloned in pBCSMH018 and pBCSMH020, upstream of the fluorescent protein gene, producing plasmids pBCSMH019 and pBCSMH021, respectively.

Construction of the plasmids encoding truncated mCherry, in which successive parts of the N-terminus of the encoded protein were removed, was obtained by ligation of the PCR products resulting from the amplification of plasmid pBCSMH001 with primer 12 combined with primers 15–18. The plasmids obtained in this way were named pBCSMH024–027.

In order to express different truncated forms of Wze-Citrine we constructed plasmids pBCSJC003, pBCSJC004 and pBCSJC005 that lacked the DNA encoding the N-terminus, central and C-terminus regions of Wze fused to Citrine, respectively. This was done by restriction and ligation of the PCR products resulting from amplification of plasmid pBCSMH004 using primer pairs 19/20, 21/22, and 23/9, respectively.

Construction of the plasmids that allowed the expression of Citrine in fusion with the first 3, 5 and 7 aminoacids of Wze at its N-terminus was carried out by ligation of the PCR products resulting from the amplification of plasmid pBCSMH004 with primer 20 combined with primers 28–30, respectively. The plasmids obtained in this way we named pBCSMJ008–010.

Construction of the plasmid pBCSJC002, which allowed the expression of Citrine in fusion with the first 50 aminoacids of Wze at its N-terminus, was carried out by ligation of the PCR products resulting from the amplification of plasmid pBCSMH004 with primer 9 combined with primer 11.

Construction of the plasmid pBCSJC007, which allowed the expression of Citrine in fusion, at its N-terminus, with the peptide sequence that in Wze is located between positions 11 to 50 was carried out by ligation of the PCR product resulting from the amplification of plasmid pBCSJC002 with primer pair 20/33.

Construction of plasmids pBCSMH030, pBCSJC001, pBCSMH031 and pBCSMH032, in which the fluorescent proteins mCherry, Citrine, CFP and GFP, respectively, are expressed in fusion with the first 10 aa of Wze, the “i-tag”, at their N-terminus, was carried out by amplification of plasmids pBCSMH003, pBCSMH004, pBCSMH019 and pBCSMH021, respectively, with primers 9 and 10, followed by restriction and auto-ligation.

In order to express Citrine in fusion, at its N-terminus, with an “i-tag” whose *wze* nucleotide sequence was modified so that it carried a silent mutation we constructed plasmid pBCSJC006. This plasmid was obtained by restriction and ligation of the PCR product resulting from amplification of plasmid pBCSJC001 using primer pair 31/32.

For expression of iCitrine-Wze and Citrine-Wze, *wze* was amplified with primers 24 and 25 and cloned into pBCSJC001 and pBCSMH002, to produce plasmids pBCSJF001 and pBCSJF002, respectively. Plasmids pBCSJF003 and pBCSJF004, which allowed the expression of iCFP-Wzd and CFP-Wzd, respectively, were constructed through amplification of *wzd* with primers 42 and 43 and cloning in plasmids pBCSMH031 and pBCSMH018, respectively. For expression of iCFP-FtsZ and CFP-FtsZ, amplification of *ftsZ* was carried out with primers 26 and 27 which was cloned into pBCSMH018 and pBCSMH031 to produced plasmids pBCSMH035 and pBCSMH036, respectively. The nucleotide sequences of the modified regions of the constructed plasmids were confirmed by sequencing. The nucleotide sequence of the plasmids pBCSJC001 and pBCSMH30-32 are available from GenBank (accession numbers KC292050 to KC292053, respectively).

### Microscopy

*S. pneumoniae* strains were grown until early exponential phase (O. D. (600 nm)  = 0.2–0.3) and observed by fluorescence microscopy on a thin layer of 1% agarose in PreC medium [24]. Images were obtained using a Zeiss Axio Observer. Z1 microscope equipped with a Plan-Apochromat objective (100×/1.4 Oil Ph3; Zeiss) and a Photometrics CoolSNAP HQ2 camera (Roper Scientific). The following Semrock filters were used to visualized the different fluorescent signals: GFP-3035B-ZHE-ZERO for GFP tagged proteins, CFP-2432A-ZHE-ZERO for CFP tagged proteins, YFP-2427A-ZHE-ZERO for Citrine tagged proteins and TXRED-4040B-ZHE-ZERO for mCherry tagged proteins. After acquisition, images were analyzed and cropped using Metamorph software (Meta Imaging series 7.5) and Image J software [26]. Fluorescence quantification was done using the Metamorph software by measuring the integrated fluorescence intensity in a defined region of 2 by 2 pixels and subtracting the minimum background fluorescence obtained from every value. The obtained values were then normalized to the higher value. Quantification was performed for at least 100 cells of each strain. Statistical analysis of the fluorescence intensity data was performed using GraphPad Prism 6 (GraphPad Software, Inc.). The nonparametric Kruskal-Wallis test, followed by Dunn's multiple comparison, was used to avoid assuming a normal distribution of the data.

### Protein analysis

Bacterial cell aliquots of 1 ml of culture were harvested at mid-exponential growth phase. Cells were incubated at 37°C during 30 minutes in deoxicholate (0.25 mg/ml), RNase (10 mg/ml), DNase (10 mg/ml) and PMSF (1 mM). For the fluorescent protein analysis, proteins were incubated with solubilization buffer (200 mM Tris-HCl pH 8.8, 20% glycerol, 5 mM EDTA pH 8.0, 0.02% bromophenol blue, 4% SDS, 0.05M DDT) [27] at 37°C during 5 minutes and separated on SDS-PAGE. Gel images were acquired on a FUJI FLA 5100 laser scanner (Fuji Photo Film Co.) with 635 nm excitation and >665 nm band pass emission filter for protein molecular weight marker detection, 532 nm excitation and >575 nm band pass emission filter for mCherry detection and 473 nm excitation and >510 nm band pass emission filter for Citrine detection.

For western-blot analysis, cells extracts were boiled during 5 minutes before being separated on SDS-PAGE. Proteins were transferred into a Hybond PVDF Membrane (Amersham) and probed with Living Colors ® Av. Peptide Antibody (Clontech) for the detection of Citrine, used at 1∶500, followed by 1∶100000 of goat anti-rabbit conjugated to horseradish peroxidase. Detection was done with ECL Plus^TM^ Western Blotting Detection Reagents (Amersham).

### RNA isolation and reverse transcriptase PCR (RT-PCR)

*S. pneumoniae* strains were grown in C+Y until early-exponential phase for RNA extraction. Prior to harvesting, RNAprotect Bacteria Reagent (twice the culture volume, QIAGEN) was added to the culture and the mixture was immediately vortexed for 10 sec. The cells were harvested, the pellet was frozen in liquid N_2_ and stored at −80°C overnight. The next day, the pellet was resuspended with 200 µl of sodium deoxycholate 0.25 mg/ml for 30 min at 37°C. RNA was extracted with RNeasy Mini kit (QIAGEN) and resuspended in milli-Q water. Total RNA was quantified using a Nanodrop Spectrophotometer ND-100. For RT-PCR, purified RNA was treated with Turbo DNase (Ambion) and screened for absence of contaminating DNA by PCR. 100 ng of DNase-treated RNA was subjected to reverse transcription using the OneStep RT-PCR Kit (QIAGEN). To amplify the fluorescent genes, the following nucleotides were used: 40/41 for citrine and 18/40 for mCherry. As a negative control, RNA isolated from strain BCSMH031 was used.

### Quantitative Real-Time PCR

cDNA was generated from 250 ng of each RNA sample using TaqMan RT Reagents (Applied Biosystems, Branchburg, NJ, USA). The reaction mix included 5.5 mM MgCl_2_, 500 µM dNTPs, 2.5 µM random hexamers, 1× RT Buffer, 0.8 U/µl RNase Inhibitor and 1.25 U/µl MultiScribe RT in a final volume of 50 µl. The Reverse Transcription conditions were 10 min at 25°C, 15 min at 42°C and 5 min at 99°C. Quantification of Citrine and mCherry expression was achieved using the ABI7000SDS (Applied Biosystems), SYBR Green chemistry, and the standard curve method for relative quantification. The PCR reagents consisted of: 1× SYBR Green PCR Master Mix (Applied Biosystems), 400 nM of each primer, and 5 µl of sample cDNA, in a final volume of 25 µl. The thermocycling profile was: 10 min at 95°C followed by 40 cycles of 15 s at 95°C and 1 min at 60°C. qPCR primers for mCherry (34 and 35) citrine (36 and 37) and tetracycline (38 and 39) were designed using ABI7000SDS – specific software, Primer Express (Applied Biosystems).

Optical plates included plasmid standard curves for Citrine and mCherry, and duplicates of each cDNA sample. “No template” and “no RT” controls were also included in every qPCR assays. For each sample, the expression of Citrine or mCherry was determined from the respective standard curve by conversion of the mean threshold cycle values, and normalization was obtained by dividing the quantity of Citrine (or mCherry) cDNAs by the quantity of cDNA amplified within the gene encoding for the tetracycline resistance protein (used as the endogenous control), which is cloned in the same plasmid. The specificity of the amplified products was verified by analysis of the dissociation curves generated by the ABI 7000 software based on the specific melting temperature for each amplicon. The final qPCR results were based on two independent experiments.

## Supporting Information

Figure S1 The fluorescence signals emitted by mCherry, Citrine and CFP Wze fluorescent derivatives do not overlap. The median fluorescence, with 25% (white error bars) and 75% (black error bars) inter-quartile range (in arbitrary units), emitted by Wze-mCherry (strain BCSMH011), Wze-Citrine (strain BCSMH012), Wze-CFP (strain BCSMH066) and Wze-GFP (strain BCSMH067) measured at each of the filters, Texas Red, YFP, CFP and GFP is plotted. At least 100 cells of each strain were quantified. Strain BCSMH052, containing an empty plasmid, was used as control. Representative images are shown at the bottom. Exposure times: Phase, 100 msec; Texas Red, YFP, CFP and GFP, 5 sec. Scale bar, 2 μm.
(TIF)

Figure S2 The presence of the i-tag does not influence the localization of the fluorescent protein. Representative pictures of the localization of proteins imCherry, iCitrine, iCFP and iGFP in the encapsulated strain ATCC6314 are shown. All proteins are dispersed throughout the cytoplasm of the cells. Exposure times: Phase, 100 msec; Texas Red, YFP, CFP and GFP, 5 sec. Scale bar, 2 μm.
(TIF)

Table S1 Bacterial strains and plasmids used in this study.
(PDF)

Table S2 Primers used in this study.
(PDF)
