# Supplementary figures and images for: Construction of Improved Tools for Protein Localization Studies in Streptococcus pneumoniae
Source: PLoS One. 2013 Jan 22;8(1):e55049. doi: 10.1371/journal.pone.0055049 (PMC3551898; doi:10.1371/journal.pone.0055049)

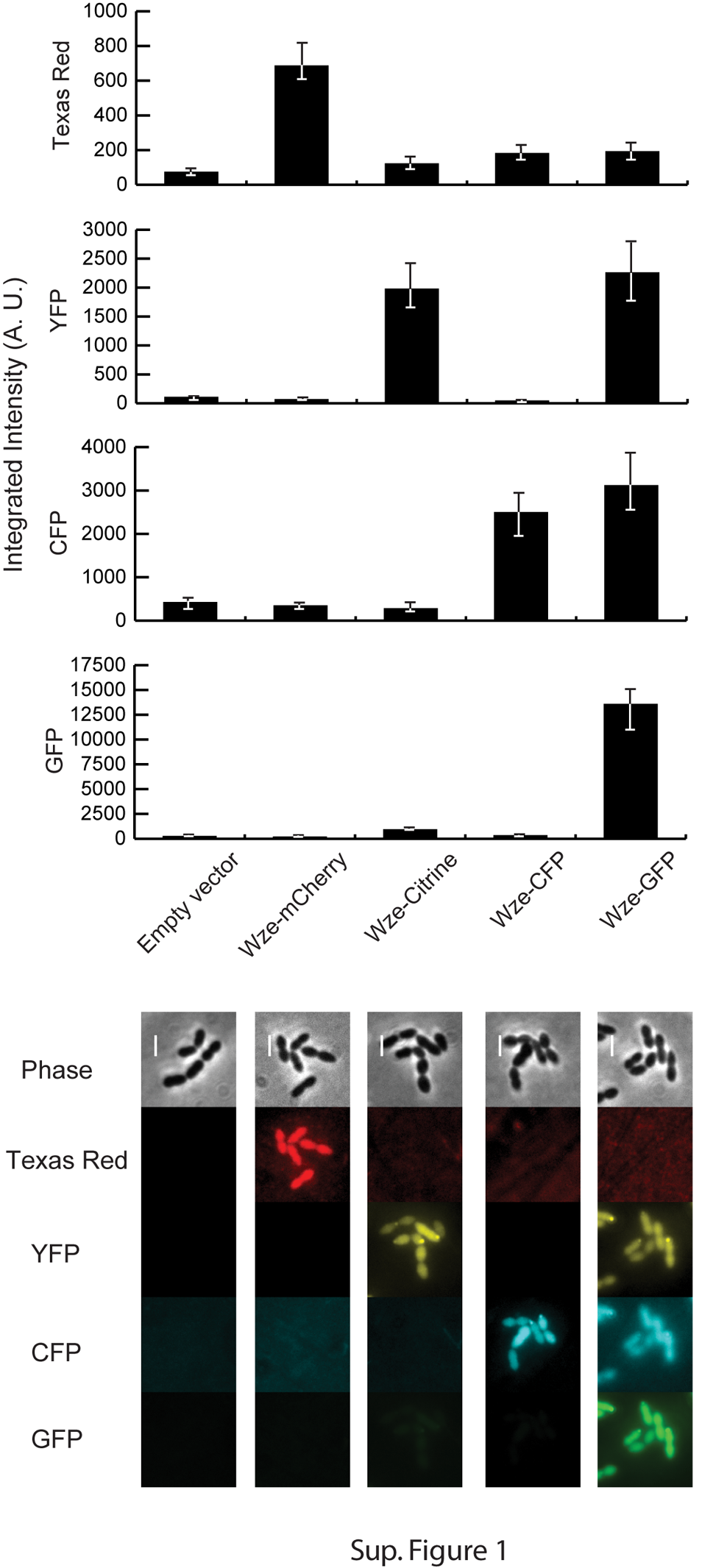

Supplement: Figure S1 — The fluorescence signals emitted by mCherry, Citrine and CFP Wze fluorescent derivatives do not overlap. The median fluorescence, with 25% (white error bars) and 75% (black error bars) inter-quartile range (in arbitrary units), emitted by Wze-mCherry (strain BCSMH011), Wze-Citrine (strain BCSMH012), Wze-CFP (strain BCSMH066) and Wze-GFP (strain BCSMH067) measured at each of the filters, Texas Red, YFP, CFP and GFP is plotted. At least 100 cells of each strain were quantified. Strain BCSMH052, containing an empty plasmid, was used as control. Representative images are shown at the bottom. Exposure times: Phase, 100 msec; Texas Red, YFP, CFP and GFP, 5 sec. Scale bar, 2 μm. (TIF) [file pone.0055049.s001.tif]

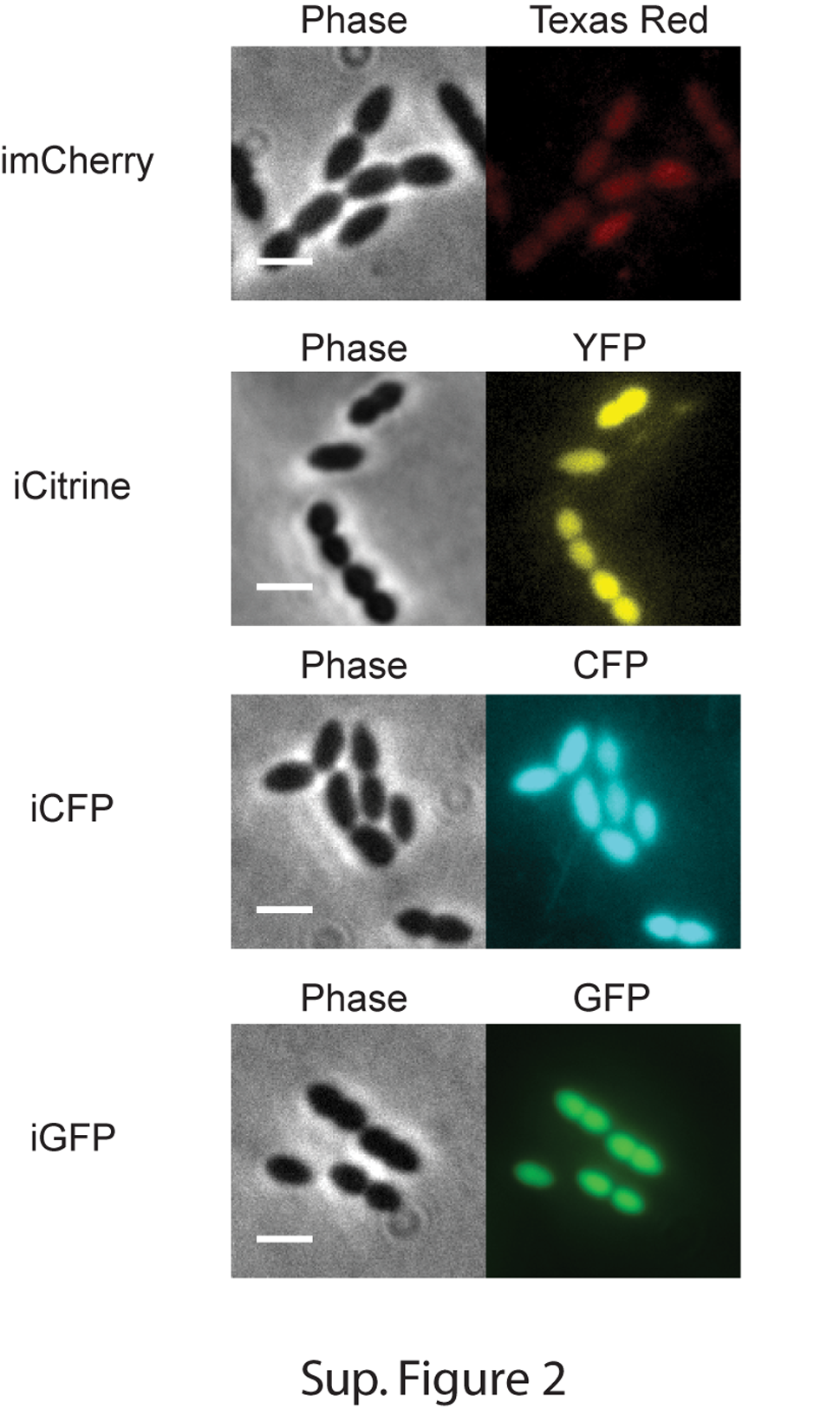

Supplement: Figure S2 — The presence of the i-tag does not influence the localization of the fluorescent protein. Representative pictures of the localization of proteins imCherry, iCitrine, iCFP and iGFP in the encapsulated strain ATCC6314 are shown. All proteins are dispersed throughout the cytoplasm of the cells. Exposure times: Phase, 100 msec; Texas Red, YFP, CFP and GFP, 5 sec. Scale bar, 2 μm. (TIF) [file pone.0055049.s002.tif]
