# Supplementary material for: Construction of Improved Tools for Protein Localization Studies in Streptococcus pneumoniae
Source: PLoS One. 2013 Jan 22;8(1):e55049. doi: 10.1371/journal.pone.0055049 (PMC3551898; doi:10.1371/journal.pone.0055049)
Supplement: Table S1 — Bacterial strains and plasmids used in this study. (PDF) [file pone.0055049.s003.pdf]

Table 1. Bacterial strains and plasmids used in this study.

Name	Relevant Characteristics	Reference
Strains		
ATCC6314	Encapsulated strain, serotype 14.	American Type Culture Collection.
BCSJC001	R36ApBCSJC001, Tet^r.	This work.
BCSJC002	R36ApBCSJC002, Tet^r.	This work.
BCSJC003	R36ApBCSJC003, Tet^r.	This work.
BCSJC004	R36ApBCSJC004, Tet^r.	This work.
BCSJC005	R36ApBCSJC005, Tet^r.	This work.
BCSJC006	R36ApBCSJC006, Tet^r.	This work.
BCSJC007	R36ApBCSJC007, Tet^r.	This work.
BCSJC008	R36ApBCSJC008, Tet^r.	This work.
BCSJC009	R36ApBCSJC009, Tet^r.	This work.
BCSJC010	R36ApBCSJC010, Tet^r.	This work.
BCSJC011	ATCC6314pBCSJC001, Tet^r.	This work.
BCSJF001	ATCC6314pBCSJF001, Tet^r.	This work.
BCSJF002	R36ApBCSJF002, Tet^r.	This work.
BCSJF003	R36ApBCSJF003, Tet^r.	This work.
BCSJF004	R36ApBCSJF004, Tet^r.	This work.
BCSMC001	ATCC6314Δ*cps*	(14)
BCSMH006	R36ApBCSMH003, Tet^r.	This work.
BCSMH007	R36ApBCSMH004, Tet^r.	This work.

BCSMH011	BCSMC001pBCSMH003, Tet^r.	(14)
BCSMH012	BCSMC001pBCSMH004, Tet^r.	(14)
BCSMH015	ATCC6314pBCSMH003, Tet^r.	(14)
BCSMH016	ATCC6314pBCSMH004, Tet^r.	(14)
BCSMH029	ATCC6314pBCSMH019, Tet^r.	This work.
BCSMH030	ATCC6314pBCSMH021, Tet^r.	This work.
BCSMH031	R36ApBCSLF001, Tet^r.	This work.
BCSMH032	R36ApBCSMH001, Tet^r.	This work.
BCSMH033	R36ApBCSMH002, Tet^r.	This work.
BCSMH034	R36ApBCSMH018, Tet^r.	This work.
BCSMH035	R36ApBCSMH019, Tet^r.	This work.
BCSMH036	R36ApBCSMH020, Tet^r.	This work.
BCSMH037	R36ApBCSMH021, Tet^r.	This work.
BCSMH040	R36ApBCSMH024, Tet^r.	This work.
BCSMH041	R36ApBCSMH025, Tet^r.	This work.
BCSMH042	R36ApBCSMH026, Tet^r.	This work.
BCSMH043	R36ApBCSMH027, Tet^r.	This work.
BCSMH045	R36ApBCSMH030, Tet^r.	This work.
BCSMH046	R36ApBCSMH031, Tet^r.	This work.
BCSMH047	R36ApBCSMH032, Tet^r.	This work.
BCSMH050	R36ApBCSMH035, Tet^r.	This work.
BCSMH051	R36ApBCSMH036, Tet^r.	This work.
BCSMH052	BCSMC001pBCSLF001, Tet^r.	This work.
BCSMH063	ATCC6314pBCSMH030, Tet^r.	This work.

BCSMH064	ATCC6314pBCSMH031, Tet^r.	This work.
BCSMH065	ATCC6314pBCSMH032, Tet^r.	This work.
BCSMH066	BCSMC001pBCSMH019, Tet^r.	This work.
BCSMH067	BCSMC001pBCSMH021, Tet^r.	This work.
R36A	Non-encapsulated laboratory strain.	Avery *et al* (1944). J Exp Med 79: 137-158
S. pneumoniae plasmids		
pBCSJC001	pBCSMH004 derivative, allowing expression of Citrine containing the first 10 aa of Wze at its N-terminus, Tet^r.	This work.
pBCSJC002	pBCSMH004 derivative, allowing expression of Citrine containing the first 50 aa of Wze at its N-terminus, Tet^r	This work.
pBCSJC003	pBCSMH004 derivative, lacking the N-terminal 147 nucleotides of *wze*, Tet^r.	This work.
pBCSJC004	pBCSMH004 derivative, lacking the central 381 nucleotides of *wze*, Tet^r.	This work.
pBCSJC005	pBCSMH004 derivative, lacking the C-terminal 147 nucleotides of *wze*, Tet^r.	This work.
pBCSJC006	pBCSJC001 derivative, TTA→CTC change in codon 4 of Wze, Tet^r.	This work.
pBCSJC007	pBCSJC002 derivative, lacking the N-terminal 33 nucleotides of Wze, Tet^r.	This work.

pBCSJC008	pBCSJC001 derivative, allowing expression of Citrine containing the first 3 aa of Wze at its N-terminus, Tet^r.	This work.
pBCSJC009	pBCSJC001 derivative, allowing expression of Citrine containing the first 5 aa of Wze at its N-terminus, Tet^r.	This work.
pBCSJC010	pBCSJC001 derivative, allowing expression of Citrine containing the first 7 aa of Wze at its N-terminus, Tet^r.	This work.
pBCSJF001	pBCSJC001 containing i*Citrine-wze*, Tet^r.	This work.
pBCSJF002	pBCSMH002 containing *Citrine-wze*, Tet^r.	This work.
pBCSJF003	pBCSMH031, containing i*CFP-wzd*, Tet^r.	This work.
pBCSJF004	pBCSMH018 containing *CFP-wzd*, Tet^r.	This work.
pBCSLF001	High-copy-number vector, contains the -10 constitutive promoter of *SigA* from *S. pneumoniae*, Tet^r.	(14)
pBCSMH001	pBCSLF001 derivative, allows expression of mCherry fusion proteins, Tet^r.	(14)
pBCSMH002	pBCSLF001 derivative, allows expression of Citrine fusion proteins, Tet^r.	(14)

pBCSMH003	pBCSMH001 containing *wze-mCherry*, Tet^r.	(14)
pBCSMH004	pBCSMH002 containing *wze-Citrine*, Tet^r.	(14)
pBCSMH018	pBCSLF001 derivative, allows expression of CFP fusion proteins, Tet^r.	This work.
pBCSMH019	pBCSMH018 containing *wze-cfp*, Tet^r.	This work.
pBCSMH020	pBCSLF001 derivative, allows expression of GFP fusion proteins, Tet^r.	This work.
pBCSMH021	pBCSMH018 containing *wze-gfp*, Tet^r.	This work.
pBCSMH024	pBCSMH001 derivative, removal of the first ten codons between the ATG and the mCherry sequence Tet^r.	This work.
pBCSMH025	pBCSMH001 derivative, removal of the first fourteen codons between the ATG and the mCherry sequence Tet^r.	This work.
pBCSMH026	pBCSMH001 derivative, removal of the first eighteen codons between the ATG and the mCherry sequence Tet^r.	This work.
pBCSMH027	pBCSMH001 derivative, removal of the twenty two codons between the ATG and the mCherry sequence, Tet^r.	This work.
pBCSMH030	pBCSMH001 derivative, expression of mCherry containing the first 10 aa of Wze at its N-terminus, Tet^r.	This work.

pBCSMH031	pBCSMH018 derivative, allowing expression of CFP containing the first 10 aa of Wze at its N-terminus, Tet^r.	This work.
pBCSMH032	pBCSMH020 derivative, allowing expression of GFP containing the first 10 aa of Wze at its N-terminus, Tet^r.	This work.
pBCSMH035	pBCSMH018 containing *cfp-ftsz*, Tet^r.	This work.
pBCSMH036	pBCSMH031 containing i*cfp-ftsz*, Tet^r.	This work.
E. coli plasmids		
pMUTIN-CFP	Plasmid containing the CFP coding sequence.	(18)
pTrc99A-GFP	Plasmid containing the GFP (P5) coding sequence.	(19)
