# Supplementary material for: Construction of Improved Tools for Protein Localization Studies in Streptococcus pneumoniae
Source: PLoS One. 2013 Jan 22;8(1):e55049. doi: 10.1371/journal.pone.0055049 (PMC3551898; doi:10.1371/journal.pone.0055049)
Supplement: Table S2 — Primers used in this study. (PDF) [file pone.0055049.s004.pdf]

Table 2. Primers used in this work.

Primer	Sequence 5' → 3'	Features/ Restriction sites
1	GGACTAGTGGGCCCGCCGGCATGGTGAGCAAGGGCG	SpeI, ApaI, NaeI
2	GAAGATCTAATCCATGGCATATGAGCGGCCGCCTTGTACAGCTCGTCC	BglII
3	GGATTAGATCTCAGGAATTG	BglII
4	CCCTTGCTCACACTAGTGCC	SpeI
5	CTAGCTAGCATGCCGACATTAGAAATAGCAC	NheI
6	CGGGGTACCTTTTTTACCATAATTTCCATAGGAAGC	KpnI
7	GGACTAGTGGGCCCGCCGGCATGAGTAAAGGAGAAG	SpeI
8	GAAGATCTAATCCATGGCATATGAGCGGCCGCGTCGACTTTGTATAGTTCATCC	BglII
9	GCGGAGCTCTGTTGCTGTTACCAAG	SacI
10	GCGGAGCTCCAGTTTTTTTGTGCTATTTC	SacI
11	GCGGAGCTCAGTTGTTTTCCTTCCCCAGG	SacI
12	GGGGTACCCATGCTAGCCTACCTCCTTAAGC	KpnI
13	GGGGTACCAGCTCGGCTGGCTCCGCTGC	KpnI
14	GGGGTACCTCCGCTGCTGGTTCTGGC	KpnI
15	GGGGTACCTCTGGCACTAGTGTGAGCAAGG	KpnI

16	GGGGTACCGTGAGCAAGGGCGAAGAAGATAAC	KpnI
17	GGGGTACCGAAGAAGATAACATGGCTATC	KpnI
18	GGGGTACCATGGCTATCATTAAAGAGTTC	KpnI
19	GCGGAGCTCACTACTTCCGTAAATATAG	SacI
20	GTAGAGCTCCATGCTAGCCTACCTCCTTAAG	SacI
21	GATGAGCTCGGTGAGGCGAATAAACGTGATG	SacI
22	GATGAGCTCTGTTTTTCCTTCCCCAGGG	SacI
23	GATGAGCTCGGTACCAGCTCGGCTG	SacI
24	ATAAGAATGCGGCCGCAATGCCGACATTAGAAATAG	NotI
25	CATGAGATCTTTATTTTTACCATAATTTCC	BglII
26	CATGCCATGGATGACATTTTCATTTGATAC	NcoI
27	GAAGGCCTTTAACGATTTTTGAAAAATGGAGG	StuI
28	GCGGAGCTCTGTCGGCATGCTAGCCTACCTCC	SacI
29	GCGGAGCTCTTCTAATGTCGGCATGCTAGCC	SacI
30	GCGGAGCTCTGCTATTTCTAATGTCGGCATGC	SacI
31	GCGGCTAGCATGCCGACACTCGAAATAGCAC	NheI
32	GCGGCTAGCCTACCTCCTTAAGCTTATTATACC	NheI
33	GCGGAGCTCGAGTTCATTAAGAAGGCAG	SacI
34	GCCATTATGACGCTGAAGTGAA	
35	TGCACAGGTTTCTTGGCTTTG	
36	GAGCTGAAGGGCATCGACTT	
37	CTTGTGCCCCAGGATGTTG	
38	AATGGTTGTAGTTGCGCGCTAT	

39	AATGCTTTACCCCTATTTTCCTTTG	
40	CCGGAATTCTTACTTGTACAGCTCGTCCATGC	
41	GGGGTACCATGGTGAGCAAGGGCGAGGAGC	
42	ATTTGCGGCCGCTCATATGATGAAGGAACAAAACAC	NotI
43	CATGCCATGGCTATTTCAACTTACTCAAG	NcoI
